# SIVA-1 interaction with PCBP1 serves as a predictive biomarker for cisplatin sensitivity in gastric cancer and its inhibitory effect on tumor growth *in vivo*

**DOI:** 10.7150/jca.92963

**Published:** 2024-06-11

**Authors:** Fan-Biao Kong, Zheng-Yi Shi, Yu-Liang Huang, Huan-Huan Chen, Qiao-Ming Deng, Kun Wu, Zhou Zhu, Lei Li, Sheng Xu, Xiao-Gang Zhong, Jian-Rong Yang, Xiao-Tong Wang

**Affiliations:** 1 Department of Colorectal and Anal Surgery, Guangxi Academy of Medical Sciences, People's Hospital of Guangxi Zhuang Autonomous Region, Institute of Minimally Invasive Technology and Applications Guangxi Academy of Medical Sciences. 6 Taoyuan Road, Nanning, Guangxi Zhuang autonomous region 530021, People's Republic of China.; 2 Department of Cosmetology and Plastic Surgery Center, People's Hospital of Guangxi Zhuang Autonomous Region, Guangxi Academy of Medical Sciences, Nanning, 530021, People's Republic of China.; 3 Department of Surgery, The First Affiliated Hospital of Guangxi University of Chinese Medicine, Nanning, Guangxi Zhuang Autonomous Region, 530023, People's Republic of China.; 4 Department of Surgery, Minzu hospital of Guangxi Zhuang Autonomous Region, Nanning, Guangxi Zhuang Autonomous Region 530001, People's Republic of China.; 5 Departments of Gastrointestinal, Hernia and Enterofistula Surgery, People's Hospital of Guangxi Zhuang Autonomous Region, Institute of Minimally Invasive Technology and Applications Guangxi Academy of Medical Sciences. 6 Taoyuan Road, Nanning, Guangxi Zhuang autonomous region 530021, People's Republic of China.; 6 Department of Hepatobiliary, Pancreas and Spleen Surgery, People's Hospital of Guangxi Zhuang Autonomous Region, Institute of Minimally Invasive Technology and Applications Guangxi Academy of Medical Sciences & Guangxi Key Laboratory of Eye Health. 6 Taoyuan Road, Nanning, Guangxi Zhuang autonomous region 530021, People's Republic of China.

**Keywords:** stomach cancer, SIVA-1, chemosensitivity, biomarker, HDRA, tumor growth

## Abstract

**Background:** SIVA-1 has been reported to play a key role in cell apoptosis and gastric cancer (GC) chemoresistance *in vitro*. Nevertheless, the clinical significance of SIVA-1 in GC chemotherapy remains unclear.

**Methods and results:** Immunohistochemistry and histoculture drug response assays were used to determine SIVA-1 expression and the inhibition rate (IR) of agents to GC and to further analyze the relationship between these two phenomena. Additionally, cisplatin (DDP)-resistant GC cells were used to elucidate the role and mechanism of SIVA-1 *in vivo*. The results demonstrated that SIVA-1 expression was positively correlated with the IR of DDP to GC but not with those of 5-fluorouracil (5-FU) or adriamycin (ADM). Furthermore, SIVA-1 overexpression with DDP treatment synergistically inhibited tumor growth *in vivo* by increasing PCBP1 and decreasing Bcl-2 and Bcl-xL expression.

**Conclusions:** Our study demonstrated that SIVA-1 may serve as an indicator of the GC sensitivity to DDP, and the mechanism of SIVA-1 in GC resistance to DDP was preliminarily revealed.

## Introduction

Gastric cancer (GC) is one of the most common malignant tumors in the digestive system. In 2023, a study of global cancer statistics showed that the number of new GC cases ranked fourth in new cases and fifth in estimated deaths among digestive cancer 1. Currently, surgery and chemoradiotherapy are the primary treatment strategies for GC. Chemotherapy plays an important role in GC treatment by shrinking the tumor or related lymph nodes, creating better conditions for surgery. However, the appearance of chemotherapeutic resistance hinders the efficacy of these drugs. The mechanism underlying chemoresistance primarily involves increased drug efflux, enhanced drug detoxification effects, enhanced DNA damage repair, promotion of apoptotic resistance and increased survival 2. Considering the complexity of the mechanisms of acquired chemoresistance, multiple genes and signaling pathways likely participate in this process. Therefore, identifying novel potential targets that can reverse chemoresistance and improve therapeutic efficacy is urgent.

Prasad *et al.* 3 screened the SIVA-1 cDNA sequence from HeLa cells and thymus cell cDNA libraries using yeast two-hybrid technology and confirmed that SIVA-1 participates in protein-protein or protein-DNA interactions, inducing apoptosis. Numerous studies have found that SIVA-1 participates in the death receptor and mitochondrial apoptosis signaling pathways to affect cell fate 4. In the NF-κB pathway, SIVA-1 inhibits the activity of IL-2 by binding to the IL-2 promoter, negatively regulating NF-κB expression to induce apoptosis 5. In addition, SIVA-1 induces apoptosis by promoting MDM2-mediated p53 degradation 4. SIVA-1 also plays a key role in cancer development. Cervical cancer research revealed that expression levels of SIVA-1 were decreased in cancer tissue, and upregulation of SIVA-1 inhibited cell proliferation and promoted apoptosis 6. Moreover, SIVA1 not only promoted the sensitivity of breast cancer cells to UV radiation and induced cell apoptosis 7 but also enhanced DDP-mediated apoptosis by activating endogenous pathways 8. Barkinge *et al.* 9 showed that low SIVA-1 expression significantly interferes with DDP-induced apoptosis in colorectal cancer cells lacking p53, suggesting that p53-mediated SIVA-1 plays an important role in DNA damage-induced apoptosis 10. These studies all suggest that SIVA-1 functions as a tumor suppressor gene in the process of cancer development by affecting apoptosis. Our previous study confirmed that upregulation of SIVA-1 reverses GC cell resistance to DDP by regulating apoptosis-related genes 11. Additionally, PCBP1 is pivotal in reversing drug resistance by directly interacting with SIVA-1 12. PCBP1, a 38 kDa RNA- or DNA-binding protein, was initially isolated from a human lymphocyte cDNA library in 1994. It exhibits ubiquitous expression across various normal tissues including bone marrow, liver, lung, kidney, and colon 13. As research delves deeper into its structure and multifaceted functions, it becomes apparent that PCBP1 primarily participates in intracellular transcription and post-transcriptional regulation 14. This involvement encompasses processes such as alternative splicing of pre-mRNA, mRNA stability, and translation. Observations across numerous cases, including cervical cancer, gastric cancer, and prostate cancer, suggest that PCBP1 may function as a tumor suppressor, impeding tumorigenesis and development 15. Furthermore, its expression levels may correlate with tumor stage and metastasis 16. However, it is unclear whether overexpression of SIVA-1 has a similar function *in vivo* as has been reported *in vitro*. Furthermore, it is unknown whether SIVA-1 expression in GC tissues correlates with drug sensitivity.

Therefore, a series of experiments were performed in this study to address these questions. First, expression of SIVA-1 in GC tissues was assessed by immunohistochemistry (IHC), and the sensitivity of GC tissues to chemotherapeutic agents was examined using a histoculture drug response assay (HDRA). Then, the correlation between SIVA-1 expression and the inhibition rate of drugs to GC tissues was examined to determine the clinical significance of SIVA-1. Finally, based on a previous study, we established a xenograft model to assess the function of SIVA-1 in GC, and apoptosis-related genes in xenograft tumors were analyzed. Collectively, our results confirmed that SIVA-1 might represent an indicator of the sensitivity of GC to DDP, playing an important role in DDP resistance in GC.

## Materials and Methods

### Patients and tissue samples

By referring to previous studies 17, a cohort of 30 GC patients who underwent surgical resection between July 2017 and December 2018 at the People's Hospital of Guangxi Zhuang Autonomous Region was included in this study. In our study, patients with gastric cancer referred to people's hospital of Guangxi between 2017 and 2018, underwent radical gastrectomy and had pathology reports. All types of gastric cancer were included into the study. Baseline characteristics regarding demographics and pathologic data included pTNM stage, tumor size, Degree of tumor differentiation, and the presence of vascular invasion were collected by reviewing the recorded files. The patients with missed incomplete or poor-quality paraffin blocks were also excluded from the study. Overall, twenty patients met the criteria. The samples were obtained through surgery as per strict inclusion-exclusion criteria.

Inclusion Criteria: ① Subjects of ages less than 70 years and more than 18 years. ② All gastric lesions showing gastric malignant tumor, on histology. ③ Subjects are willing to participate in follow-up.

Exclusion Criteria: ① Subjects positive for HIV or HPV antibody. ② Subjects who have history of drug intake such as Chinese herbal medicine. ③ Subjects associated with malnutrition or with other diseases like Tuberculosis / diabetics / hypertension / typhoid / emphysema / autoinflammatory diseases. ④ Subjects with symptom of Alzheimer's disease. ⑤ Subjects suffering leukemia/ colorectal cancer / ovarian cancer / cervical cancer / breast cancer or lung cancer. ⑥ Subjects undergoing neoadjuvant chemotherapy for gastric cancer.

Each case of fresh GC tissue was separated into two parts: one was utilized for HDRA, and the other was used for IHC assays. In addition, the postoperative treatment and survival status of these 30 patients were followed up until December 2021.The Ethics Committee of the People's Hospital of Guangxi Zhuang Autonomous Region approved this research (No. KY-GZR-2022-095), and all patients provided written informed consent.

### IHC staining

To determine the expression levels of SIVA-1 in GC tissues, an IHC assay was performed following the standard streptavidin-peroxidase (SP) method described previously 18. Briefly, tumor tissues were cut into 4 μm section that were baked at 60 °C for 1 h and subsequently dehydrated in gradient ethanol solutions. The tumor sections were incubated with anti-SIVA1 antibodies (cat. Ag25748, Proteintech, Wuhan, China., diluted at 1:200) in a humidified environment overnight at 4 °C. Human small intestine tissue served as positive control. Phosphate buffer solution (PBS) served as a negative control. All processes were conducted according to the manufacturer's instructions. The IHC score of each section was examined by two independent pathologists. The IHC score is composed of the strength and the percentage of the stained positive cells. The strength evaluation criteria of positive cells were negative (no staining, 0 points), weak (light yellow, 1 point), medium (dark yellow, 2 points), and strong (brownish yellow, 3 points). The percentage of positive cells was 0 for less than 1%, 1 for 1-25%, 2 for 26-50%, 3 for 51-75%, and 4 for 76-100%. The IHC score was calculated as the strength score multiplied by the percentage score and was defined as (-) for 0-2 points, (+) for 3-6 points, and (++) for more than 6 points.

### Histoculture drug response assay

HDRA was used to determine the inhibition rate of the drug to tumor tissues as previously described 19, 20. Fresh cancerous tissues were cut into fragments of approximately 10 mg in weight and then placed into 24-well plates containing drugs dissolved in culture medium and 10% fetal bovine serum. The control group contained no drug. Six samples were used for each group. The plates were incubated in a humidified atmosphere containing 5% CO_2_ at 37 °C for 7 days. Subsequently, 3-(4,5-dimetylthiazol-2-yl)-2,5-diphenyl tetrazolium bromide (MTT) solution (5 mg/mL, 100 μl) and type I collagenase solution (0.1 mg/mL, 100 μl) were added to each well and incubated with tumor tissue for another 8 h. Then, dimethyl-sulfoxide (DMSO) was employed to extract and dissolve the MTT formazan. The absorbance value of the solution in each well was measured at 540 nm using a microplate analyzer. The inhibition rate (IR) of drug to tumor tissue was calculated using the following formula: (1-T/C) *100%, where T is the absorbance value within 1 gram of GC tissue fragment in the experimental group and C is the absorbance value within 1 gram of GC tissue fragment in the control group. Drug resistance was defined as an inhibition rate of less than 30%. Drug sensitivity was defined as an inhibition rate of more than or equal to 30%, and greater than 50% was considered highly sensitive. The concentration of drugs used in the HDRA was determined according to the peak plasma concentration in the human body: DDP was 20 μg/mL, 5-fluorouracil (5-FU) was 300 μg/mL, and adriamycin (ADM) was 15 μg/mL 21, 22. All drugs were purchased from Shandong Qilu Pharmaceuticals.

### Cell culture and transfection

The GC cell line MKN45/DDP, which resists DDP, was obtained from the medical laboratory of People's Hospital of Guangxi Zhuang Autonomous Region 11. Cell culture medium consisted of Roswell Park Memorial Institute (RPMI)-1640 medium (Procell, Wuhan, China), 10% fetal bovine serum (Thermo Fisher, Waltham, MA, USA), 100 U/mL penicillin G, 100 mg/mL streptomycin and 0.8 μg/mL DDP. The cells were maintained in a humidified atmosphere with 5% CO_2_ at 37 °C.

A SIVA-1 lentiviral vector was used to transfect MKN45/DDP cells to alter SIVA-1 expression. Transfection was performed as previously described 11. The pGV358-GFP plasmid was used to construct the SIVA-1 vector, and the SIVA-1 DNA sequence was obtained from Ensembl (ID: ENSG00000184990). Cells in logarithmic growth phase were counted and plated at 5×10^5^ cells per well in 6-well plates. Lipofectamine 2000 (Invitrogen, Carlsbad, CA, USA) was used for transfection according to the manufacturer's instructions. The cells were collected for further experiments 48 h after transfection. The MKN45/DDP cells were divided into three groups. The cells that were not transfected were considered the control group. Cells transfected with LV-NC were considered the LV-control group, and those transfected with LV-SIVA-1 were defined as the LV-SIVA-1 group.

### Xenograft experiments

The Animal Ethics Committee of the People's Hospital of Guangxi Zhuang Autonomous Region approved these animal experiments. The *in vivo* experiments complied with the Laboratory Animal Guideline for Ethical Review of Animal Welfare of the People's Republic of China. A total of 15 female BALB/c nude mice (5 weeks old) were purchased from Vital River Company (Beijing, China) and reared in the Laboratory Animal Center of Guangxi Medical University (Nanning, China). The laboratory provided an SPF feeding environment, with adequate and clean water and food daily, and gentle movements to relieve pain during cell and drug injections. Different groups of MKN45/DDP cells (2×10^6^/mouse) were subcutaneously injected into the left anterior flank of nude mice to establish the xenograft model. The health and behavior of nude mice was monitored every day. When xenografts were visible, a Vernier calipers were used to measure the length and width of tumors every 4 d, and DDP solution was intraperitoneally injected (injection dose: 3mg/Kg). Tumor volume was calculated using the following formula: length×width^2^×0.5 cm^3^. All the 15 nude mice were sacrificed by cervical spondylolysis when the volume of any xenograft was more than 1500 mm^3^ on day 13 after injection of cells. When the voluntary activity of nude mice disappeared after cervical spondylolysis, xenografts were then dissected and harvested for further analysis.

### Western blot analysis

Xenografts in the same group were mixed, and then RIPA lysis buffer (Beyotime, Shanghai, China) was used to extract total protein from the xenografts in each group. The BCA Protein Quantification kit (Beyotime) was used to quantify total protein content. Proteins were denatured by boiling for 5 min and were then separated by SDS-PAGE (Beyotime). Then, PVDF membranes (Millipore, USA) were used to transfer the proteins. Next, 5% defatted milk was employed to block the membranes for 1 h at approximately 20 °C. Primary antibodies against SIVA-1 (cat. No. 12532, Cell Signaling Technology, USA, diluted at 1:1,000), Bcl-2 (cat. No. 4223, Cell Signaling Technology, 1:1,500), Bcl-XL (cat. No. 2762, Cell Signaling Technology, 1:1,500), PCBP1 (cat. No. 8534, Cell Signaling Technology 1:500), and GAPDH (cat. No. 5174, Cell Signaling Technology, 1:1,000) were incubated with the membranes at 4°C overnight. The next day, TBST was employed to rinse the membranes, and then the HRP-labeled secondary antibody (1:10,000, Cell Signaling Technology) was used to label the membranes at room temperature (approximately 20 °C) for 1 h. The membranes were exposed to ECL reagent (Beyotime), imaged and quantitatively analyzed using an Odyssey instrument (LI-COR Biosciences). GAPDH served as the internal control.

### TdT-mediated dUTP nick-end labeling (TUNEL) assay

Apoptotic cells in sections of xenograft tissues were detected by the TUNEL apoptosis detection kit (Roche, Switzerland) according to the manufacturer's instructions. All sections from each group were assessed under a microscope (Olympus, Tokyo, Japan) in ten random nonredundant fields (scale bar: 100 μm). Brown-stained cells were defined as apoptosis-positive. The apoptosis index was calculated using the following formula: apoptosis index= number of apoptosis-positive cells/total number of cells× 100%.

### External validation

Twenty patients diagnosed with gastric cancer and treated with neoadjuvant chemotherapy at the First Affiliated Hospital of Hebei North University in January 2024 were included in the study, following the same criteria for inclusion and exclusion as previously described. Each patient underwent three cycles of neoadjuvant chemotherapy (XELOX regimen). On the first day of treatment, patients received intravenous oxaliplatin injection (from Nanjing Pharmaceutical Factory Co., Ltd., with State Pharmaceutical Approval Character H20000686) diluted in 500 mL of 5% dextrose solution, administered via continuous infusion over 2 hours at a dose of 130 mg/m2. From days 1 to 14 of the chemotherapy cycle, patients took oral capecitabine tablets (from Chia Tai Tianqing Pharmaceutical Group Co., Ltd., with China National Pharmaceutical License H20143044) at a dose of 1000 mg/m2, twice daily. Following completion of the chemotherapy regimen, patients had a 7-day rest period before starting the next cycle, with each cycle lasting 21 days. Evaluation of the short-term chemotherapy response was conducted based on solid tumor evaluation criteria 23. Complete remission (CR) was defined as complete disappearance of lesions, absence of new lesions, normalization of tumor marker levels, and maintenance of this status for ≥4 weeks. Partial response (PR) was defined as a reduction of at least 30% in the sum of the largest diameters of lesions, with maintenance of this status for ≥4 weeks. Stable disease (SD) was defined as a reduction of less than 30% or an increase of less than 20% in the sum of the largest diameters of lesions. Progressive disease (PD) was defined as the appearance of new lesions or an increase of more than 20% in the sum of the largest diameters of lesions. Chemotherapy outcomes were categorized as CR, PR, or SD (indicating effectiveness) and PD (indicating ineffectiveness). All patients completed three cycles of neoadjuvant chemotherapy and were followed up for at least 4 weeks post-treatment.

### Statistical analysis

The data are presented as the means ± standard deviation (SD). Spearman's rank correlation test was used to assess the correlation between SIVA-1 levels and inhibition rate of chemotherapeutic agents to paired tumor tissues. Differences between two groups were analyzed using Student's t tests. LSD and SNK method for one-way ANOVA was used to compare significant differences between multiple groups. P values less than 0.05 were considered statistically significant.

## Results

### Expression of SIVA-1 in GC is positively correlated with the IR of DDP to GC

**Figure 1:**
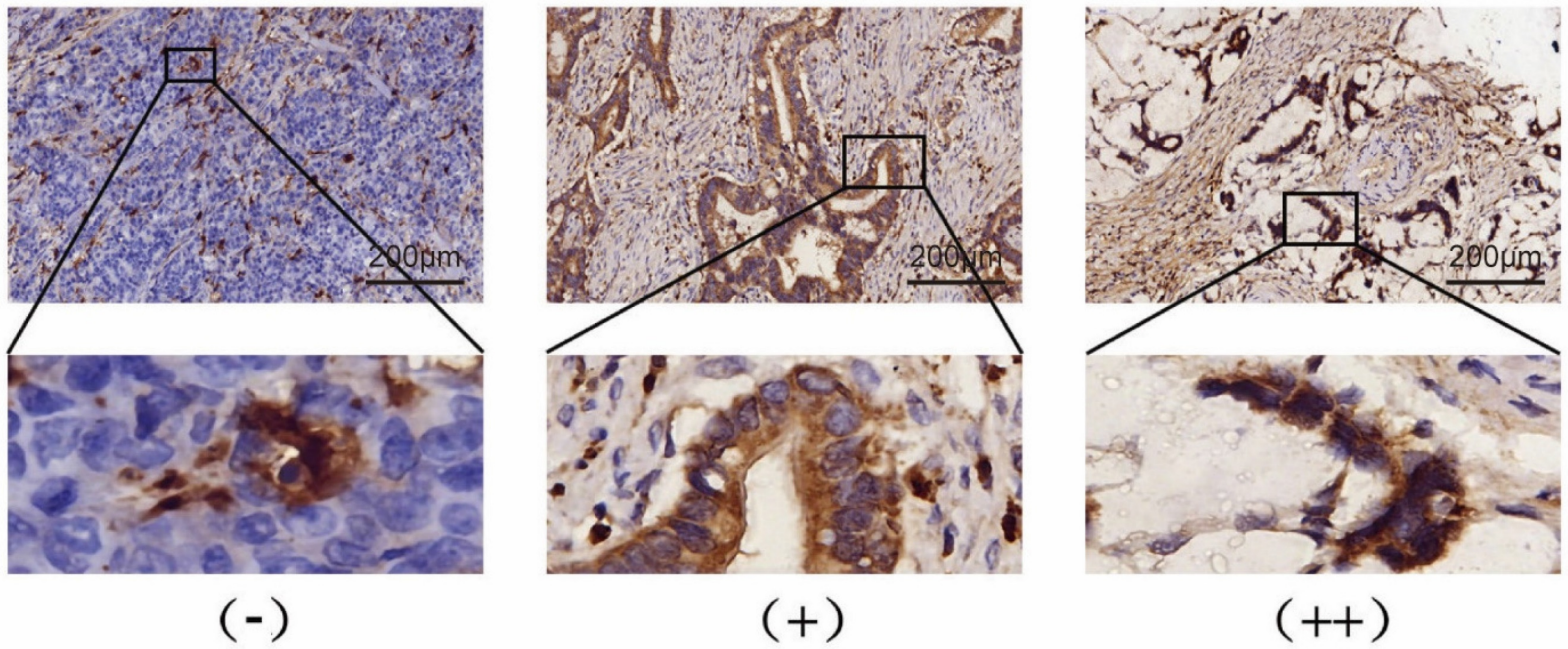
SIVA-1 (yellow or brown) is expressed in the cytoplasm of gastric cancer cells. Protein expression levels of SIVA-1 in gastric cancer tissues detected by IHC. IHC staining for SIVA-1: scale bar: 200μm.

**Figure 2:**
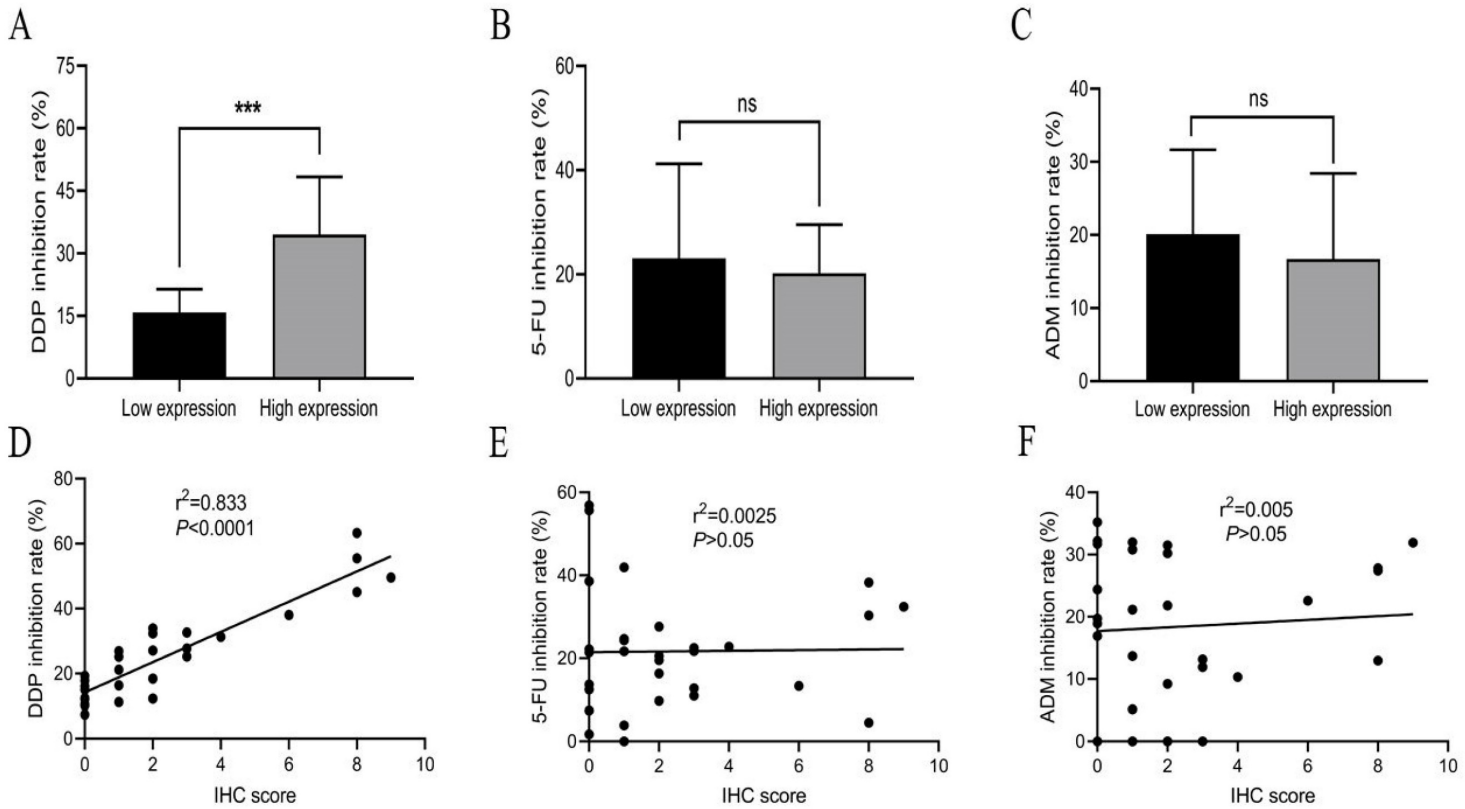
Expression of SIVA-1 is positively correlated with the IR of DDP in GC but not with that of 5-FU or ADM. A-C. The IR of drugs in the relatively high expression and relatively low expression groups. D-F. The relationship between protein expression levels of SIVA-1 in GC tissues and IR of drugs in GC. ns: no significance. *** P < 0.001. Data are shown as the mean ± SD.

Expression levels of SIVA-1 in GC tissues, which were determined by IHC assay, are shown in Table 1 and Fig. 1. According to the median IHC score, the GC cohort was divided into two groups, those with relatively low expression and those with relatively high expression (Supplementary material 1). The HDRA method was utilized to determine the inhibition rates of DDP, 5-FU and ADM in GC tissues. Then, the rate of sensitivity of cancerous tissues to each drug was calculated. As shown in Table 2, fresh GC sections displayed differential sensitivities and IRs to each drug. In order of high to low, the inhibition rates of drugs to GC were DDP, 5-FU, and ADM. The correlation between the expression of SIVA-1 and the IR of each drug to GC was then analyzed. The results indicated that expression levels of SIVA-1 in GC were positively correlated with the IR of DDP to GC. However, there was no significant relationship between SIVA-1 expression and the IR of the other two drugs in GC (Fig. 2). Among these 30 patients, 4 patients could not be contacted, 2 patients did not receive adjuvant DDP after surgery, 4 patients did not receive postoperative adjuvant chemotherapy and the remaining 20 patients have received the follow up of 3-year disease-free survival (DFS) and overall survival (OS). The results showed that there was no statistically significant difference in 3-year DFS and OS between the relatively high and relatively low SIVA-1 expression groups (Table 3).

### Overexpression of SIVA-1 cooperates with DDP to inhibit GC proliferation *in vivo*

**Figure 3:**
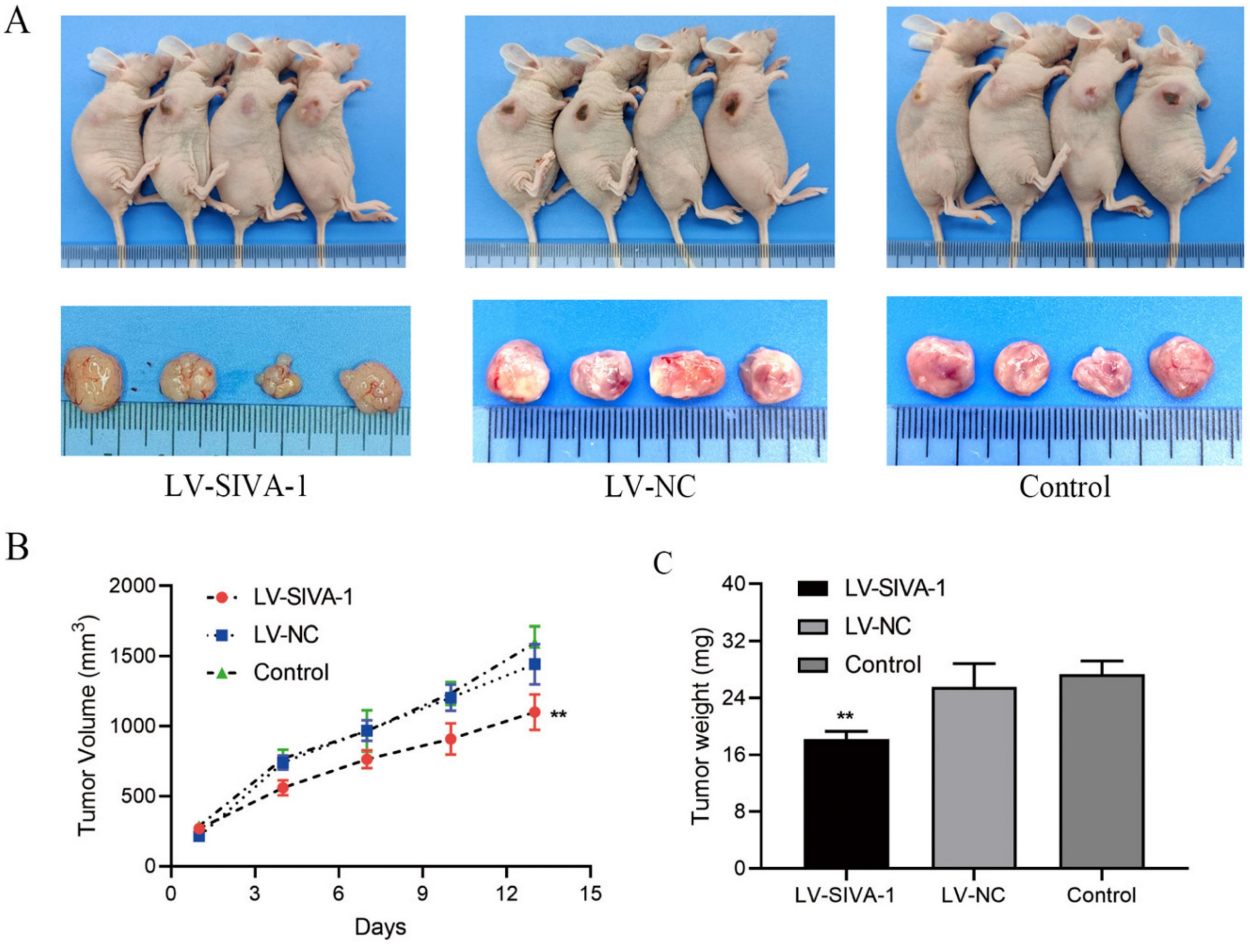
Overexpression of SIVA-1 cooperates with DDP to suppress the proliferation of GC cells *in vivo*. A. The tumor size of xenograft model in different groups of nude mice were measured. B. A xenograft growth curve was plotted based on continuous measurement of tumor volume every 4 days. C. The average tumor weights from different groups were determined. ** P < 0.01. Data are presented as the mean ± SD.

In the xenograft experiments, DDP-resistant GC MKN45/DDP cells in different groups were subcutaneously injected into nude mice to construct the xenograft model. At the same time, each nude mouse received intraperitoneal injection of DDP every 4 d. Tumor volume was calculated to examine whether SIVA-1 reverses GC cell resistance to DDP and inhibits cell growth *in vivo*. After subcutaneous injection, xenografts from mice in each group were continuously measured for 13 days, and a growth curve was created. The growth curve and the tumor weight of xenografts in each group demonstrated that overexpression of SIVA-1 effectively enhanced the sensitivity of GC cells to DDP and inhibited tumor growth *in vivo* (Fig. 3).

### Upregulation of SIVA-1 cooperates with DDP to induce GC apoptosis *in vivo*

**Figure 4:**
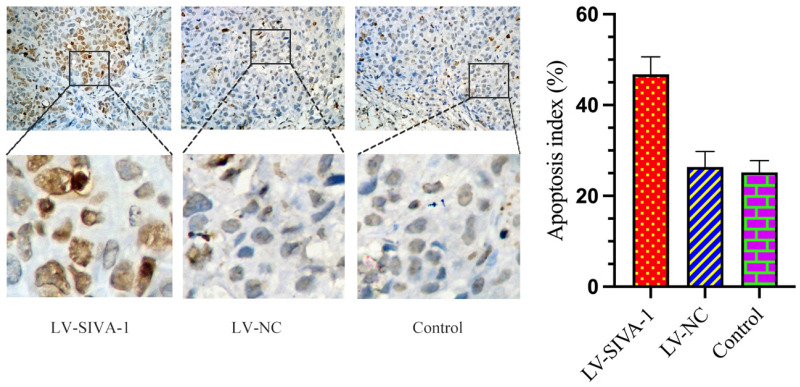
Upregulation of SIVA-1 cooperates with DDP to induce GC apoptosis *in vivo*. The TUNEL method was used to examine apoptotic cells in xenograft tumor tissues from different groups. TUNEL staining for apoptotic cells: scale bar: 200 μm. ** P < 0.01. Data are shown as the mean ± SD.

The TUNEL assay was utilized to determine whether SIVA-1 overexpression promotes DDP-induced apoptosis. As shown in Fig. 4, there were many more brown cells in the LV-SIVA-1 group than in the LV-NC and control groups. The apoptosis index in the LV-SIVA-1 group was also higher than that in the LV-NC and control groups.

### Overexpression of SIVA-1 decreases the expression levels of Bcl-2 and Bcl-xL and increases those of PCBP1

**Figure 5:**
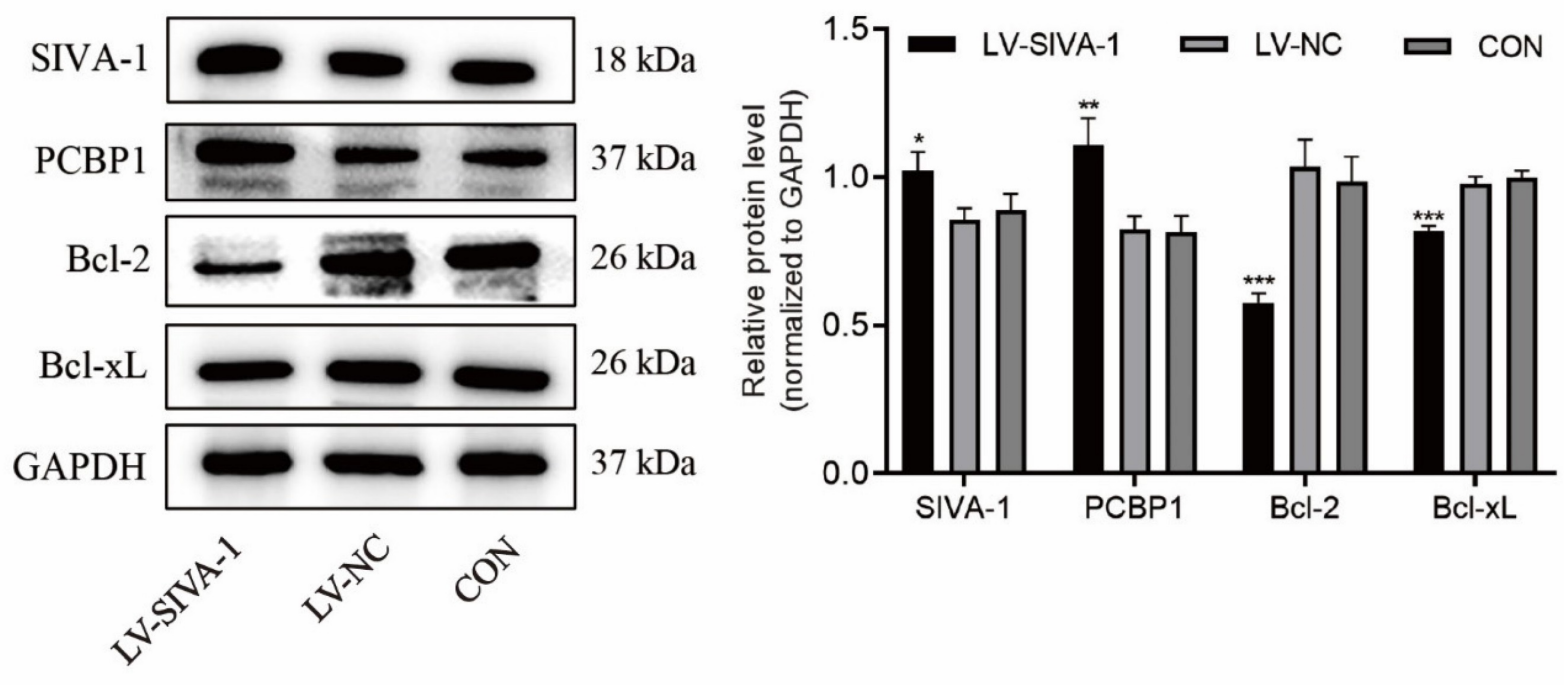
Upregulation of SIVA-1 increases levels of PCBP1 and decreases expression of Bcl-2 and Bcl-xL. Xenografts in the same group were mixed for extraction of total protein, and then apoptosis-related genes, including PCBP1, Bcl-2 and Bcl-xL, were detected by western blot. *P < 0.05 and ** P < 0.01. The independent experiments were repeated three times, and the data are shown as the mean ± SD.

To further explore the underlying mechanism by which SIVA-1 regulates DDP resistance in GC, the expression levels of Bcl-2, Bcl-xL and PCBP1 in xenograft tissues were examined by immunoblotting. Compared to the LV-NC and control groups, Bcl-2 and Bcl-xL levels were far lower, while PCBP1 levels were much higher in the LV-SIVA-1 group (Fig. 5).

**Figure 6:**
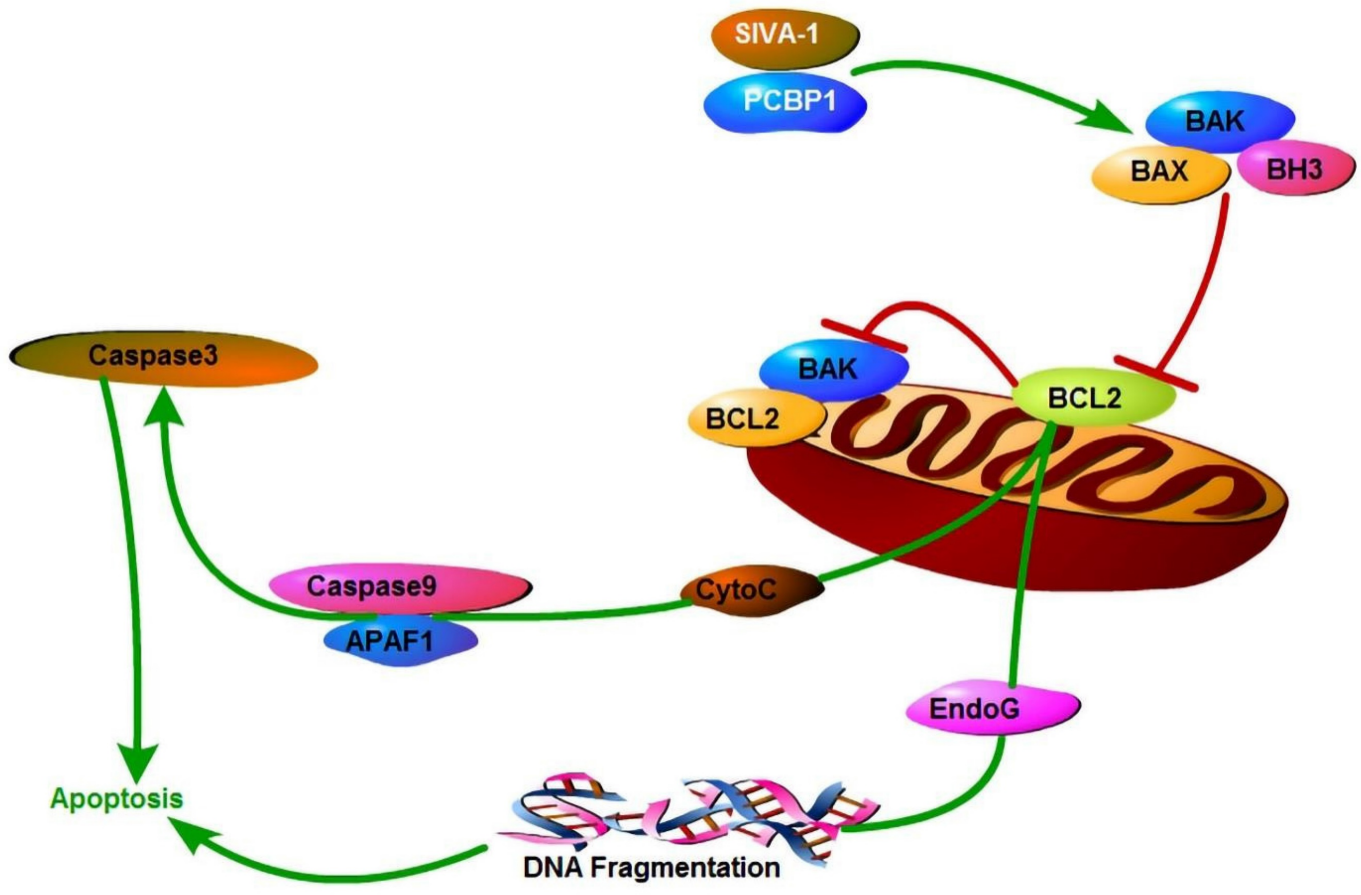
Mechanism model of SIVA-1 promoting apoptosis in gastric cancer. SIVA-1 interacts with PCBP1 to inhibit the expression and activity of Bcl-2, and then activates Caspase3/9 and finally leads to cellular apoptosis in human GC cells.

Taken our previous studies 11,18 and the results of the present study together suggested that SIVA-1 inhibited the expression and activity of Bcl-2 and activated Caspase3/9 by binding PCBP1, and finally promoted the cellular apoptosis of GC cells (Fig. 6).

### External validation of the effectiveness of SIVA-1 as a predictive biomarker for cisplatin sensitivity in gastric cancer

Among the 30 gastric cancer patients studied, 9 exhibited high siva-1 expression, with 8 showing responsiveness to neoadjuvant chemotherapy and 1 exhibiting resistance. In contrast, 21 patients displayed low siva-1 expression, with 10 responding favorably to neoadjuvant chemotherapy and 11 showing resistance. The efficacy rate of neoadjuvant chemotherapy in patients with high siva-1 expression (88.89%) was markedly higher than that in the low expression group (47.62%), with a statistically significant difference (p<0.05) (Supplementary material 2).

## Discussion

Chemotherapy has played a crucial role in the treatment of advanced GC over the past few decades 24. However, multidrug resistance (MDR) has gradually become a major clinical problem, leading to inefficient chemotherapy and poor prognosis in patients with GC 25. Although numerous new therapeutic options are available, individual variability in drug sensitivity remains a challenge. Therefore, identifying novel biomarkers that may predict the response to chemotherapy and further understanding the underlying mechanism of MDR are urgently needed. In the present study, we performed HDRA to examine the inhibition rate of chemotherapy drugs in GC tissues and analyzed the relationship between the expression of SIVA-1 and drug IRs to GC tissues. Our results revealed that SIVA-1 expression was positively correlated with the IR of DDP to GC but not with that of ADM or 5-FU. Overexpression of SIVA-1 reversed DDP resistance and inhibited the proliferation of GC cells *in vivo*. More importantly, SIVA-1 improved DDP sensitivity in GC by promoting the expression of PCBP-1 and silencing Bcl-2 and Bcl-xL. Our previous results demonstrated that SIVA-1 expression was decreased in GC tissues 18 and was even lower in MKN45/DDP cells than in MKN45 cells (unpublished data). Collectively, SIVA-1 may serve as a predictor of DDP sensitivity and a potential sensitizer in GC DDP chemotherapy.

To date, there are several methods for detecting and estimating tumor sensitivity to chemotherapy, including the HDRA. HDRA was first introduced by Hoffman et al 26. Compared to other tests, such as *in vitro* isolated tumor cell culture or *in vivo* xenograft models, the advantages of HDRA include its low cost, rapid timeline, and convenience. More importantly, HDRA is a three-dimensional system, enabling the structure of the cell-cell and cell-substrate to be maintained, and drug delivery is simulated under physical conditions 27,28. Previous studies have shown that HDRA exhibits relatively high sensitivity and specificity in the response of clinical chemotherapy to different types of cancer, with accuracy of approximately 74-92.1% 29, 30, 31, 32. Therefore, in this study, we selected the HDRA system to determine the inhibition rate of various drugs to GC tissues. Our results confirmed the operability of HDRA and suggested that the inhibition rate of DDP was higher than that of either ADM or 5-FU. Nevertheless, the IR% of DDP in our study was much lower than that in a previously published article on GC 33. One possible reason for this discrepancy is individual variability in the included samples. A larger sample cohort is needed to further research this issue. When combined with RT-qPCR or IHC, HDRA could be an effective way to screen for biomarkers with predictive capability for certain chemotherapy agents. For pemetrexed and related sensitivity, expression of TS in colorectal cancer could serve as a predictor 34. Previous research also demonstrated that expression levels of hENT-1, MT and ERCC1 could be regarded as molecular biomarkers for predicting the sensitivity of cholangiocarcinoma to gemcitabine and DDP 17. Additionally, the SULF2 methylation levels are negatively correlated with DDP sensitivity in GC and represent a potential prognostic molecular marker for GC patients treated with chemotherapy involving platinum drugs 33. In our study, expression levels of SIVA-1 were determined by IHC; subsequently, the correlation between SIVA-1 levels in GC and the inhibitory rate of chemotherapy drugs against GC was analyzed. We found that SIVA-1 expression was positively correlated with DDP IR. However, the expression of SIVA-1 in GC was not significantly correlated with IR of 5-FU or ADM to GC. This might be related to the different pharmacological mechanisms involved in SIVA-1. These results demonstrate that SIVA-1 may be a potential predictor for DDP sensitivity in GC chemotherapy.

Subsequently, we further analyzed the relationship between SIVA-1 expression in GC and clinical outcomes of patients who underwent DDP adjuvant chemotherapy. From the limited data analysis results, it seems that SIVA-1 relative high expression in IHC have better tumor outcomes than that low expression. However, there was no statistical difference between the two groups, so we need to conduct prospective studies to expand the sample size and extend the follow-up time to obtain more convincing evidence in the further study.

Many studies have confirmed that SIVA-1 in combination with DDP synergistically promotes tumor cell apoptosis through a variety of mechanisms. In p53-deficient colorectal cancer cells, low expression of SIVA1 significantly hindered DDP-induced apoptosis 9. In HeLa cells, degradation of the TXA2 receptor was blocked by the binding of SIVA-1 to the C-terminus of the TXA2 receptor, and endogenous accumulation of the TXA2 receptor stimulates and promotes apoptosis induced by DDP 35. In addition, our previous study demonstrated that overexpression of SIVA-1 increases the sensitivity of GC DDP-resistant cells to DDP and promotes apoptosis induced by DDP 11. However, the results in our previous study showed that upregulation of SIVA-1 increases the tolerance of vincristine (VCR)-resistant GC cells to VCR and promotes proliferation and migration by regulating the NF-κB signaling pathway 18. These studies suggest that SIVA-1 appears to play distinct roles in the response to different chemotherapeutic agents. In this study, we revealed that upregulation of SIVA-1 cooperates with DDP to inhibit tumor growth and promote MKN45/DDP cell apoptosis *in vivo* by increasing levels of PCBP1 and decreasing expression of Bcl-2 and Bcl-xL. Poly (rC)-binding proteins (PCBPs), a distinct subset within the RNA binding protein family, are distinguished by their strong affinity and specific interaction with poly-cytosine (poly-C) sequences 36. The PCBP family consists of five members, which include hnRNP K (heterogeneous nuclear ribonucleoprotein K) along with PCBP1 through PCBP4 37. These proteins share a similar structural motif, featuring triple hnRNP K homology (KH) domains crucial for recognizing and binding C-rich regions within mRNA as well as single- and double-stranded DNA. Numerous investigations have highlighted the significant involvement of PCBPs in various cellular processes such as growth, differentiation 38, and tumorigenesis 39, exerting their influence across multiple levels of regulation. The results of the current study further confirmed that SIVA-1 cooperates with DDP in the treatment of GC.

Consistent with our previous study, we found that PCBP1 is a direct target of SIVA-1 in mRNA gene chip, a yeast two-hybrid experiment and the Co-IP has confirmed this result (unpublished data). The current study also confirmed that PCBP1 was significantly regulated as determined by SIVA-1 overexpression. PCBP1 is widely expressed in human lung, liver, colon, and other normal tissues and is expressed at low levels in many kinds of primary and metastatic cancers, such as GC 40, prostate cancer 15 and hepatocellular carcinoma 41. Increasing evidence has confirmed that PCBP1 functions as a tumor suppressor that regulates gene expression at multiple levels, including transcription 42, and translation 43. A previous study discovered that loss of PCBP1 inactivates caspase-3 and PARP-1, leading to apoptosis disorder 44. More importantly, upregulation of PCBP1 might induce apoptosis by reducing the expression of Bcl-2 45 and inhibiting Bcl-xL expression levels by targeting alternative splicing of STAT3 exon 23 46. In terms of mechanisms about apoptosis signal pathway, there may be an indirectly regulatory relationship between PCBP1 and apoptosis. Overexpression of PCBP1 not only inhibited autophagy of cancer cells, but also reduced the expression of anti-apoptotic proteins Bcl-2, then triggered cancer cells apoptosis mediated by caspase-3 and caspase-8 45. Additionally, PCBP1 regulates AKT activation to mediate oxaliplatin resistance in colorectal cancer 47. As mentioned above, PCBP1 not only plays a positive role in cancer cell apoptosis but also in inhibiting chemotherapy resistance. Our results revealed that the potential mechanism of SIVA-1 involves enhancing the therapeutic effect of DDP and promoting apoptosis by modulating the expression of PCBP1 in GC.

In conclusion, the current study demonstrated that SIVA-1 represents a predictive biomarker of sensitivity to DDP treatment in GC, and SIVA-1 acts as a tumor suppressor that reverses the resistance of gastric cancer cells to DDP by regulating apoptosis-related molecules. This study is helpful further understand the role and potential mechanism of SIVA-1 in DDP resistance in GC. The crucial role of SIVA-1 in chemotherapy for GC may represent a promising therapeutic target for overcoming chemotherapy resistance. Based on the present research, we will further concentrate on analyzing the relationship between SIVA-1 expression and the response of GC patients to DDP-based chemotherapy in the future. Additionally, downregulation of SIVA-1 and the upstream regulatory mechanism of SIVA-1 should be conducted and explored to comprehensively expound upon its function and mechanism in multidrug resistance in GC.

## Supplementary Material

Supplementary tables.

## Figures and Tables

**Table 1:** Expression of SIVA-1 in gastric cancer tissues assessed by IHC.

			Protein expression	
Gene	Cases	(-) (cases)	(+) (cases)	(++) (cases)
SIVA-1	30	20	6	4

IHC: Immunohistochemical.

**Table 2:** Inhibition rate of chemotherapy drugs on gastric cancer tissues examined by HDRA.

Drug	Cases	Resistant (cases)	Sensitive (cases)	Highly- sensitive (cases)	Sensitivity (%)	Inhibition rate (%) (median)	Inhibition rate (%) (mean±sd)
DDP	30	21	8	2	30	23.16	25.2±14.03
5-FU	30	23	6	1	23.33	21.54	21.66±14.24
ADM	30	22	8	0	26.67	19.72	18.41±11.55

HDRA: Histoculture Drug Response Assay; DDP: Cisplatin; 5-FU: 5-Fluorouracil; ADM: Adriamycin.

**Table 3:** Correlation between SIVA-1 expression in gastric cancer and clinical outcome of patients received adjuvant chemotherapy with DDP.

Group	Cases	3-year DFS (%)	3-year OS (%)
High expression	12	83.33 (10/12)	91.67 (11/12)
Low expression	8	75.00 (6/8)	87.50 (7/8)
*X^2^* value		0.21	0.09
*P* value		0.65	0.76

DFS: Disease-free survival, OS: Overall survival
