# Supplementary material for: SIVA-1 interaction with PCBP1 serves as a predictive biomarker for cisplatin sensitivity in gastric cancer and its inhibitory effect on tumor growth in vivo
Source: J Cancer. 2024 Jun 11;15(13):4301–12. doi: 10.7150/jca.92963 (PMC11212092; doi:10.7150/jca.92963)
Supplement: Supplementary file 1 — Supplementary tables. [file jcav15p4301s1.pdf]

Supplemental Material 1

Table 4 Comparative Analysis of Siva-1 Positive Expression Rate among Gastric Cancer Patients with Various Clinicopathological Characteristics.

Clinicopathological features	n	SIVA-1 High expression	SIVA-1 low expression	X^2 value	P value
Age (year)					
≤60	16	3	13	0.403	0.526
>60	14	4	10		
Sex					
Male	21	4	17	2.078	0.149
Female	9	4	5		
Tumor size (cm)					
≤3	12	5	7	2.301	0.129
>3	18	3	15		
Degree of tumor differentiation					
Moderately differentiated	9	2	7	0.13	0.719
Poorly differentiated	21	6	15		
Lymph node metastasis					
Yes	22	4	18	0.17	0.68
No	8	2	6		
Intravascular cancer thrombus					
Yes	16	3	13	1.099	0.295
No	14	5	9		
Neuroaggression					
Yes	17	4	13	0.197	0.657
No	13	4	9		
Tumor staging					
I&II	11	3	8	0.151	0.698
III&IV	19	4	15		

Supplemental Material 2

Table 5: Relationship between the expression levels of siva-1 detected by IHC and the efficacy of neoadjuvant chemotherapy in gastric cancer.

Neoadjuvant chemotherapy	n	SIVA-1 High expression	SIVA-1 low expression	P value
Effective	18	8	10	0.049
Ineffective	12	1	11	
Total	30	9	21	

IHC: Immunohistochemical.
